# Fluvastatin Mediated Breast Cancer Cell Death: A Proteomic Approach to Identify Differentially Regulated Proteins in MDA-MB-231 Cells

**DOI:** 10.1371/journal.pone.0108890

**Published:** 2014-09-30

**Authors:** Anantha Koteswararao Kanugula, Vishnu M. Dhople, Uwe Völker, Ramesh Ummanni, Srigiridhar Kotamraju

**Affiliations:** 1 Centre for Chemical Biology, CSIR-Indian Institute of Chemical Technology, Hyderabad, India; 2 Interfacultary Institute of Genetics and Functional Genomics, University Medicine Greifswald, Greifswald, Germany; King Faisal Specialist Hospital & Research center, Saudi Arabia

## Abstract

Statins are increasingly being recognized as anti-cancer agents against various cancers including breast cancer. To understand the molecular pathways targeted by fluvastatin and its differential sensitivity against metastatic breast cancer cells, we analyzed protein alterations in MDA-MB-231 cells treated with fluvastatin using 2-DE in combination with LC-MS/MS. Results revealed dys-regulation of 39 protein spots corresponding to 35 different proteins. To determine the relevance of altered protein profiles with breast cancer cell death, we mapped these proteins to major pathways involved in the regulation of cell-to-cell signaling and interaction, cell cycle, Rho GDI and proteasomal pathways using IPA analysis. Highly interconnected sub networks showed that vimentin and ERK1/2 proteins play a central role in controlling the expression of altered proteins. Fluvastatin treatment caused proteolysis of vimentin, a marker of epithelial to mesenchymal transition. This effect of fluvastatin was reversed in the presence of mevalonate, a downstream product of HMG-CoA and caspase-3 inhibitor. Interestingly, fluvastatin neither caused an appreciable cell death nor did modulate vimentin expression in normal mammary epithelial cells. In conclusion, fluvastatin alters levels of cytoskeletal proteins, primarily targeting vimentin through increased caspase-3- mediated proteolysis, thereby suggesting a role for vimentin in statin-induced breast cancer cell death.

## Introduction

Emerging data suggest that the pleotropic effects of statins (HMG-CoA reductase inhibitors) contribute to their anti neoplastic, anti inflammatory and neuroprotection. *In vitro*, preclinical and clinical data shows that lipophilic statins are effective against various cancers including breast cancer [1]–[3]. Recently, Nielsen et al., reported that the mortality rate is significantly decreased in prostate cancer patients treated with statins compared to non-statin users [4]. Lipophilic statins inhibit breast cancer cells (ER positive, HER2 positive and ER negative) growth by decreasing NF-kB, AP-1 activation and phosphorylation of MAPK [5]. Previously, we have shown that both fluvastatin and simvastatin induce MCF-7 breast cancer cell death by inducing nitric oxide *via* iNOS and arginase dependent pathways [6]. Also, recently we reported that fluvastatin and simvastatin induce triple negative breast cancer (TNBC) cell death by increasing iNOS-dependent nitric oxide levels and dys-regulation of iron homeostasis in MDA-MB-231, MDA-MB-453 and BT-549 cells [7]. Statins are known to deplete mevalonate pathway intermediates including the synthesis of isoprenyl groups necessary for activating the Rho/Ras/Rac GTPases that play a significant role in cancer cell proliferation and invasion. Though statins are known to inhibit cholesterol biosynthesis through mevalonate pathway, they may target multiple proteins regulating different pro survival pathways thereby inhibiting proliferation of cancer cells. Aka et al., recently compared a functional proteome of two hormone-dependent breast cancer cells lines MCF-7 and T47D and the analyses showed that 164 proteins involved in various proliferative functions are differentially expressed between them [8]. Lovastatin induces breast cancer cell death through modulation of E2F1-pathway by altering expression of prohibitin and retinoblastoma (Rb) proteins [9]. Upon exposure to lovastatin in ARO thyroid cancer cells, a set of proteins were altered in their expression which were then mapped to various cellular functions related to protein folding, metabolism, signal transduction, protein expression and protein degradation [10]. Isobaric tags for relative and absolute quantitation (iTRAQ)-based proteome analysis of ZR-75-1 and MDA-MB-231 breast cancer cells treated with chemotherapeutic agent doxorubicin followed by death receptor ligand TRAIL revealed perturbation of various pathways including cellular assembly and organization, molecular transport, oxidative stress, cell motility and cell death. Further, this study also identified three proteins (PPIB, AHNAK, and SLC1A5) that are commonly regulated in both the cell types upon the drug exposure [11]. Recently, stable isotope labeling by/with amino acids in cell culture -based proteomic approach in lovastatin-induced human acute promyelocytic leukemia (HL-60) cells quantified 3200 proteins, among which 120 proteins were significantly altered which were mapped to regulating various cellular pathways including inhibition of cholesterol biosynthesis, estrogen receptor signaling, glutamate metabolism and protein ubiquitination [12].

In the present study, we investigated the comparative proteome of metastatic MDA-MB-231 breast cancer cells exposed to fluvastatin and control treated cells by 2-D gel electrophoresis (2-DE) for protein separation followed by LC-ESI-MS/MS for protein identification. The differentially expressed proteins were analysed by gene ontology and Ingenuity Pathway Analysis (IPA) to understand the molecular functions of proteins and pathways regulated by fluvastatin. The major hubs of significant sub networks and their non canonical pathways were validated by western blot analysis. This systematic analysis revealed the involvement of various signaling networks in determining their key role in mediating fluvastatin-induced MDA-MB-231 cell death. Taken together, this study surmises a newer means of statin induced cancer cell death identifying a set of proteins which may serve as prediction markers up on further validation to monitor fluvastatin treatment in breast cancer patients.

## Materials and Methods

### Reagents, cell lines and culture conditions

Fluvastatin, caspase-3 inhibitor (Boc-D-FMK) and MG-132 were purchased from Calbiochem. Dulbecco's modified eagles medium (DMEM), Dulbecco's phosphate buffered saline (DPBS), cholera toxin, mevalonolactone, trypan blue, urea, thiourea, CHAPS, DTT, idoacetamide and sodium dodecyl sulphate were purchased from Sigma chemicals and foetal bovine serum (FBS) was from Lonza. IPG linear strips (pH 4–7, 11 cm) and IPG phor buffer were procured from GE healthcare life sciences. BOC-Leu-Arg-Arg-Arg-AMC was purchased from Biomol and all other chemicals used were of reagent grade. All cell lines were purchased from ATCC. MDA-MB-231 and BT-549 (breast adenocarcinoma cell lines) cells were grown in 10% DMEM containing 10% FBS, L-glutamine (4 mmol/L), penicillin (100 units/mL), and streptomycin (100 µg/mL). MCF-10A (non-tumorigenic epithelial cells) were grown in MEBM supplemented with BPE, hEGF, insulin, hydrocortisone and cholera toxin (100 ng/mL). Cells were maintained in an incubator at 37°C under humidified atmosphere with constant supply of 5% CO_2_.

### Preparation of protein extracts from cells

MDA-MB-231 and MCF-10A cells were treated with fluvaststain (10 µM) in triplicate for a period of 24 h and at the end of the treatment, cells were washed with ice cold DPBS and collected in PBS by cell scraper. Cells were centrifuged at 3000 rpm for 5 minutes at 4°C. Cell pellets were directly lysed in an appropriate volume of 2D-lysis buffer (8 M Urea, 2 M Thiourea, 4% CHAPS, 65 mM DTT, 40 mM Tris-HCl) and homogenized for 60 seconds under ice cold conditions. To remove cell debris, lysates were centrifuged at 12000 rpm for 10 minutes at 4°C. Clear supernatants were collected and protein concentrations were measured using Bradford reagent.

### Two-dimensional gel electrophoresis

For two-dimensional gel electrophoresis, isoelectric focussing (IEF) was performed using linear pH 4–7 (11 cm) IPG strips (GE Healthcare) according to the manufacturer's instructions with minor modifications to improve resolution. Briefly, 250 µg protein extracts prepared as described above were diluted to 125 µl with rehydration buffer (8 M urea, 2 M thiourea, 2% CHAPS, 50 mM DTT with 0.5% v/v IPG buffer pH 4–7, (GE Healthcare) and used to passively rehydrate each IPG strip overnight. Proteins were separated by the Ettan IPG Phor 3 (GE) at 20°C. The focusing was started at 250 V for 15 minutes, 500 V for 60 minutes and then increased to 1000 V with 60 minutes and 6000 V to 9000 Vh linear gradient. The samples were maintained at 6000 V until a total run of 12 kVh. After IEF, the IPG strips were equilibrated (10 minutes) in equilibration buffer (0.375 M Tris-HCl, pH 8.8, 6 M urea, 20% glycerol, 2% SDS and 130 mM DTT) and then transferred to equilibration buffer containing 135 mM iodoacetamide (10 minutes) with constant shaking. Equilibrated strips were applied onto the top of 12% SDS-PAGE gels and sealed with 1% agarose prepared in SDS-Tris-glycine buffer with trace amounts of bromophenol blue as a tracking dye to monitor electrophoresis. Electrophoresis was performed at a constant current of 25 mA per gel. At the end of electrophoresis, proteins were visualized by staining gels with colloidal coomassie brilliant blue staining solution (Roth Chemicals, Germany). Briefly, gels were fixed in 40% methanol, 15% acetic acid for 1 h and then soaked with colloidal staining solution overnight. Gels were then de-stained in solution containing 10% methanol, 7% acetic acid to remove background interference.

### Image analysis

Colloidal coomassie brilliant blue stained gels were scanned at 300 dpi resolution using PROPIC II (DIGILAB, Genomic solutions). For the identification of differentially expressed proteins, Image analysis was carried out with PDQUEST 2-D analysis software package (Version 6.0, Bio-Rad). In a match set for comparative image analysis, gels consisting of triplicate treatments were grouped accordingly and analysed for qualitative and quantitative differences. To ensure that the differences in spot volume and density between gels are due to differential expression, all the gels were normalized using total spots density normalization tool. The minimum fold changes between groups was restricted to greater than 1.7 fold and ANOVA test was performed for statistical significance (p<0.001) of differentially altered spots across the gel members in a match set.

### Protein identification by LC-MS/MS

Preparation of peptide mixtures for protein identification was performed as described previously [13]. Briefly, selected protein spots were excised manually from Colloidal Coomassie Brilliant Blue stained 2-DE gels and trypsin digestion was performed manually. Gel pieces were destained by washing with 50% (v/v) of 50 mM ammonium bicarbonate/acetonitrile and dehydrated with acetonitrile. After drying, gel pieces were soaked in ammonium bicarbonate solution and then in trypsin solution (20 ng/µl trypsin in 20 mM ammoniumbicarbonate) till the gel pieces were submerged and incubated at 37°C for 15 h. The digestion was stopped by the addition of 5% acetic acid. For peptide extraction, gel pieces were covered with 50% (v/v) ACN/0.1% (w/v) acetic acid and incubated for 30 minutes at 37°C with constant shaking. The supernatant was collected in designated tubes before repeating the extraction procedure. The peptide extracts were pooled into respective tubes and dried in speed vac and reconstituted in buffer with 0.1% acetic acid containing 2% acetonitrile prior to the measurement in the FT-ICR instrument.

### Mass spectrometry

Proteins identification from peptide mixtures was performed on LTQ-Fourier transform-ion cyclotron resonance (FT-ICR) mass spectrometer (Thermo Scientific, Germany) equipped with nano electrospray ion source coupled to a nanoACQUITY Ultra Performance LC (UPLC) (Waters Corporation, USA) and controlled by Xcalibur software (Thermo Electron Corporation, Gemany). Briefly, peptides were enriched on a nanoAcquity Symmetry C18 pre-column (2 cm length, 180 µm inner diameter and 5 µm particle size) from Water Corporation and separated using NanoAcquity BEH130 C18 column (10 cm length, 100 µM inner diameter and 1.7 µm particle size from Water Corporation) on nanoAcquity UPLC. The peptides were eluted with a mixture of solvent gradient (1–5% buffer B in 2 min, 5–60% buffer B in 23 min, 60–99% buffer B in 3 min of buffer A (2% acetonitrile containing 0.1% acetic acid) and B (acetonitrile containing 0.1% acetic acid). The eluted peptides were electro sprayed into mass spectrometer. The mass spectrometric data was collected in data dependent mode to switch between FT-ICR MS and LTQ-MS/MS acquisition automatically. Full scan MS spectra in the m/z range from 300 to 1500 (resolution r = 50000) were acquired in the mass spectrometer. The data acquisition method was set to isolate up to five most intense ions depending upon signal intensity for fragmentation in the linear ion-trap using collision induced dissociation. Target ions already selected for MS/MS were dynamically excluded for 30 seconds. The general conditions of mass spectrometer for data collection were 1.6–1.7 kV electrospray voltage, Ion selection threshold 1000 counts for MS/MS. For identification of proteins from MS data, automated database search was performed using Proteome Discoverer 1.3.0.339 (Thermo Scientific, Germany) with Sequest algorithm. A human uniprot fasta database and a decoy database of 1% FDR were used for identification. Two possible missed cleavages for trypsin enzyme specificity with a mass tolerance of 10 ppm (parent ion) and 0.8 Da (fragment ion), methionine oxidation as dynamic were considered for data base search. The identity of the proteins was confirmed based on high confidence peptide identification containing at least two peptides per protein with rank 1 peptides in proteins and XCorr scores ≥2.25 or 2.75 for doubly and triply charged peptides respectively.

### Bioinformatics analysis of the proteomic data

Differential expression data were analysed by unsupervised hierarchical clustering to find unique protein clusters among the altered proteins and sample clusters based on the identified proteins. The clustering was performed using Euclidean distance measure from the log-transformed values of fold change in expression of all significant differentially regulated proteins across samples included in the analysis set. The differentially expressed proteins and their respective biological processes and molecular functions or relationships were determined using Database for Annotation, Visualization, and Integrated Discovery (DAVID) GO (gene ontology) annotation tool. Further, to map protein networks to which differentially regulated proteins are mapped, the lists of all differentially regulated proteins were entered into the IPA software to assess their interaction with other proteins and relevant biological networks. A global master network connecting all differentially expressed proteins was constructed based on annotations published in literature. This network shows most relevant direct and indirect connections of proteins found to be deregulated between fluvastatin treated and untreated MDA-MB-231 cells. From the larger non canonical networks, further sub networks were built to focus activated/inactivated pathways. Based on the inter connectivity of the objects (identified proteins) and edges with in the sub network, major hubs were identified for further validation to understand their role in fluvastatin-induced MDA-MB-231 cell death.

### Reverse Transcription-PCR analysis

Following the treatment, total RNA was extracted with 1 mL of TRIzol reagent (Sigma) according to the manufacturer's protocol. First-strand cDNA synthesis was made using five micrograms of RNA according to the manufacturer's instructions (Takara blue print 1^st^ strand cDNA synthesis kit). One microliter of the cDNA mixture per gene was used to amplify the respective genes. Primer sets for GAPDH (forward-5′GCACCACCAACTGCTTAGCAC3′, reverse-5′CCCTGTTGCTGTAGCCAAAT3′) and vimentin (forward-5′ GCCTCTCCAAAGGCTGCAGA3′; reverse-5′GAGTTTTTCCAAAGATTTAT3′).

### Western blotting

Cells were washed with ice-cold DPBS and resuspended in 150 µL of radioimmune precipitation assay (RIPA) buffer containing 1 mmol/L of sodium vanadate, 10 µg/mL of aprotinin, 10 µg/mL of leupeptin and 10 µg/mL of pepstatin inhibitor. Cells were homogenized by passing the suspension through a 25-gauge needle (20 strokes). The lysate was centrifuged at 750 rpm for 10 min at 4°C to pellet out the nuclei. The remaining supernatant was centrifuged for 30 min at 12000 rpm. Total protein was determined using the Bradford method. Proteins were resolved using 10% SDS-PAGE and blotted onto nitrocellulose membranes. Membranes were washed with TBS (140 mmol/L NaCl, 50 mmol/L Tris-HCl; pH 7.2) containing 0.1% Tween-20 (TBST) and 5% skimmed milk to block nonspecific protein binding. Membranes were incubated with 1 µg/mL of rabbit anti-vimentin polyclonal antibody, rabbit anti-β-catenin antibody (Cell Signaling Technology) and rabbit anti-GAPDH antibody (Sigma) in TBST for overnight at 4°C, washed five times with TBST, and then incubated with goat anti-rabbit IgG horseradish peroxidase–conjugated secondary antibody (1∶5000) for 1.5 h at room temperature. Immunoreactive proteins were detected using the enhanced chemiluminescence method (Amersham Biosciences).

### Trypan blue exclusion cytotoxicity assay

MDA-MB-231 and MCF-10A cells were treated with various concentrations of fluvastatin (0–20 µM) for a period of 48 h and also various time points (0–48 h) with fluvastatin (10 µM). Also, cells were incubated with fluvastatin in the presence or absence of mevalonolactone (25 µM) for a period of 48 h and at the end of the treatment, cells were washed with DPBS and trypsinized. Cell viability/death was measured by trypan blue dye exclusion assay using a Countess automated cell counter (Invitrogen).

### Proteasome function assay: 26S proteasome

MDA-MB-231 cells were incubated with various concentrations of fluvastatin (0–20 µM) and also for different time periods (0–48 h). At the end of the treatments, cells were washed with buffer I (50 mM Tris, pH 7.4; 2 mM DTT; 5 mM MgCl_2_; 2 mM ATP) and homogenized with buffer I containing 250 mM sucrose. Twenty micrograms of 10000 rpm supernatant was diluted with buffer I to a final volume of 900 µl. The fluorogenic proteasome substrate BOC-Leu-Arg-Arg-AMC (trypsin-like) was added at a final concentration of 80 µM. Proteolytic activity was measured by monitoring the release of the fluorescent group 7-amido-4- methylcoumarin (excitation 380 nm, emission 460 nm) [14].

### Statistical Analysis

Data are expressed as Mean ± SD and statistically analyzed by the two-tailed, unpaired, Student's t-test and scores were considered significant when p<0.05.

## Results

### Fluvastatin induces cytotoxicity in MDA-MB-231 breast cancer cells: Effect of mevalonate

**Figure 1:**
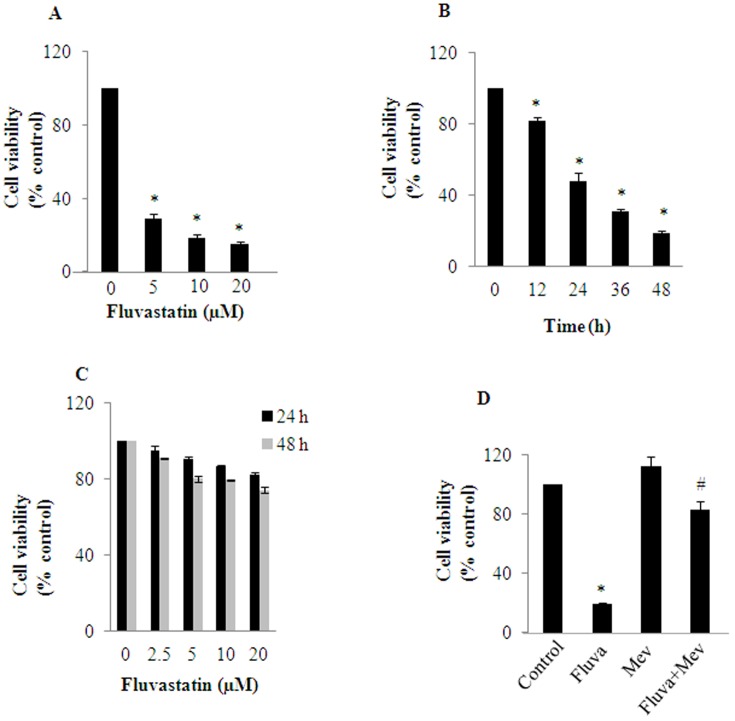
Fluvastatin induces cell death in MDA-MB-231 breast cancer cells: Effect of mevalonate. A, MDA-MB-231 cells were treated with various concentrations of fluvastatin (0–20 µM) for 48 h and B shows cells treated for different time points with 10 µM fluvastatin. At the end of the treatments, cell viability was measured by trypan blue exclusion method. C, MCF-10A cells were treated with fluvastatin (0–20 µM) for a period of 24 and 48 h and cell death was measured as described earlier. D, MDA-MB-231 cells were treated with fluvatstatin (10 µM) in presence or absence of mevalonate (25 µM) for a period of 48 h and cell viability was measured by trypan blue exclusion assay. Data represented is Mean±SD from at least three independent experiments. *, significantly different compared to untreated conditions; #, significantly different compared to mevalonate and fluva+mev condition. Statistical significance was tested at P<0.05 level by two-tailed, unpaired, Student's t-test.

Initially, fluvastatin-induced cytotoxicity was measured as a function of dose (0–20 µM) and time (0–48 h) in MDA-MB-231 cells by trypan blue exclusion assay. Fluvastatin both dose and time dependently increased MDA-MB-231 cell death (Fig. 1A and B). Fluvastatin at 10 µM caused around 50% cell death by 24 h in MDA-MB-231 cells but whereas in MCF-10A cells, fluvastatin did not cause an appreciable cell death (Fig. 1C). The IC_50_ of fluvastatin in MCF-10A cells for 24 h was found to be 50 µM. Furthermore, the effect of mevalonate, an immediate downstream product of HMG-CoA was assessed on fluvastatin-induced MDA-MB-231 cell death. Cell were treated with 10 µM fluvastatin in the presence or absence of mevalonate (25 µM) for a period of 48 h and found that fluvastatin-induced cytotoxicity was significantly inhibited in the presence of mevalonate (Fig. 1D). Thereby suggesting that, fluvastatin-mediated anti-proliferative effects are as a result of inhibition of mevalonate pathway intermediates (Fig. 1D).

### Proteomic analysis of Fluvastatin treated MDA-MB-231 breast cancer cells

**Figure 2:**
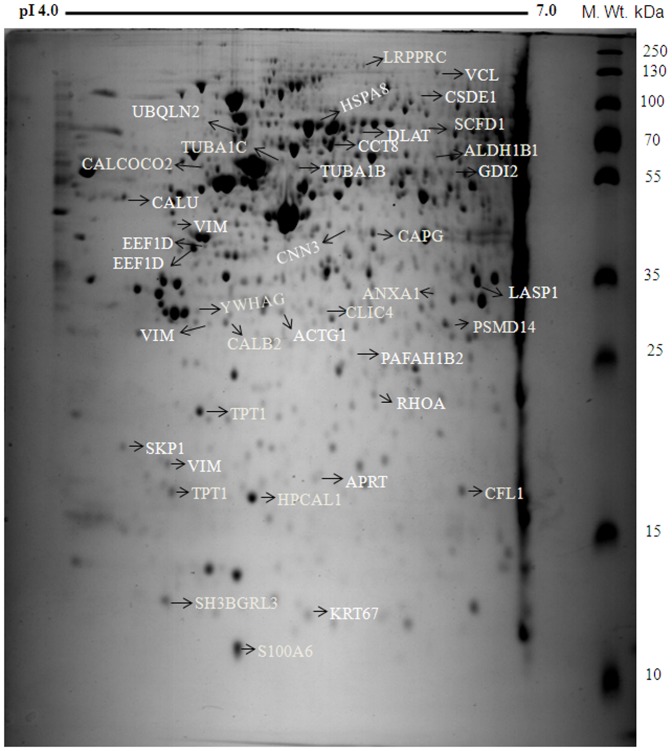
Proteome map of fluvastatin regulated proteins in MDA-MB-231 breast cancer cells. MDA-MB-231 cells were either untreated or treated with fluvastatin (10 µM) for a period of 24 h and then both untreated and treated cell lysates were subjected to iso-electric focusing and second dimension was resolved with 12% SDS-PAGE. Differentially regulated protein spots were indicated and marked. Gel shown is a representative of three independent experiments.

**Figure 3:**
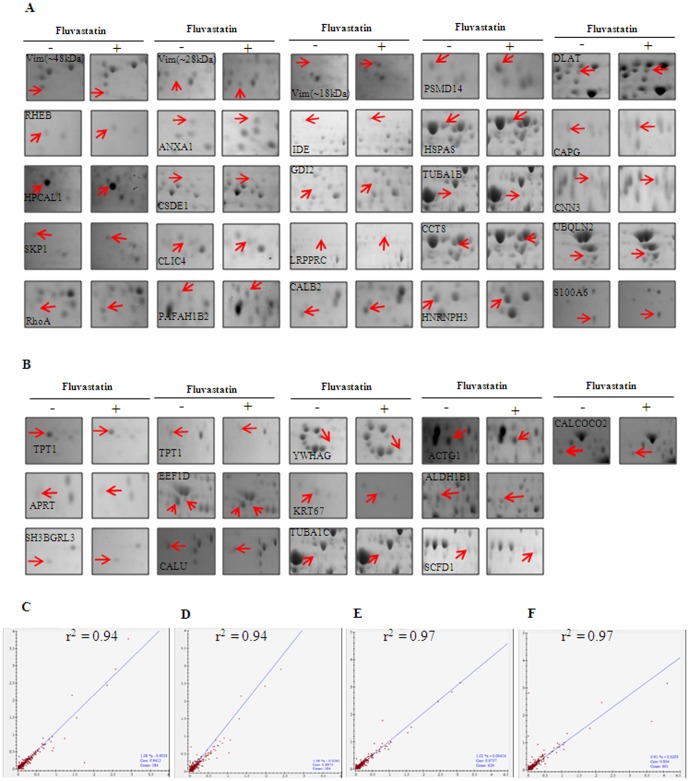
Enlarged view of fluvastatin mediated differentially regulated proteins of the differentially regulated proteins in MDA-MB-231 cells. A and B shows enlarged view of statin mediated up regulated (A) and down regulated (B) spots respectively. The results presented in C–F are from 3 independent experiments. C and D represent the correlation coefficient of untreated MDA-MB-231 cell lysate. C represents the comparison between gel 1 versus gel 2 and D represents gel 1 versus gel 3. E and F represent the correlation coefficient of statin treated MDA-MB-231 cell lysate. E represents the comparison between gel 1 versus gel 2 and F represents gel 1 versus gel 3.

In this study we analyzed the differentially regulated protein profiles in MDA-MB-231 metastatic breast cancer cells treated with fluvastatin. For this, cells were treated with fluvastatin (10 µM) for 24 h and protein extracts were prepared according to the procedure mentioned in the methods section. Proteins were resolved using 2D SDS–PAGE in a pH range of 4–7 with a molecular weight range between 10 and 150 kDa. With this approach, about 800 protein spots per gel were detected with more sensitive colloidal comassie staining (Fig. 2). The protein expression patterns were compared using the PDQUEST software for quantitative and qualitative differences. The 2-DE image analysis employed for the identification of proteins from protein spots of altered expression revealed 39 spots with differential expression in MDA-MB-231 cells treated with fluvasatin. Among the identified proteins, 25 proteins displayed increased levels and 14 proteins displayed reduced levels in fluvastatin treated cells compared to untreated conditions. After in-gel digestion, these proteins were identified by LC-MS/MS. The list of altered proteins and the degree of differential expression is presented in Table 1. From these identified proteins, we found more than one spot containing the same protein identity with different molecular weight and pI, which may be due to protein fragmentation induced by fluvastatin treatment. A selection of differentially expressed proteins both up regulated and down regulated are presented in Fig. 3 A and B respectively as enlarged gel images. To further confirm the reproducibility of the observed differences between the triplicate gels within each group, we have performed the correlation coefficient analysis using PDQUEST software. The results showed that the correlation coefficient between the untreated cell lysate gels was 0.94 (Fig. 3C and 3D) and between the fluvastatin treated cell lysate gels was 0.97 (Fig. 3E and 3F).

**Table 1:** Identification of proteins from differentially regulated protein spots by mass spectrometry using LTQ-FTICR.

Protein name	Accession	MW (kDa)	pI	C/T fold change	Protein score	Sequence coverage (%)	No. of peptides matched	Protein function (www.uniprot.org)
S-phase kinase-associated protein 1	P63208	18.6	4.54	1.76	2493.75	71.17	11	Essential component of the SCF (SKP1-CUL1-F-box protein) ubiquitin ligase complex, which mediates the ubiquitination of proteins involved in cell cycle progression, signal transduction and transcription.
Keratin, type II cytoskeletal 6B	P04264	55.5	8.59	1.93	6465.28	44.72	29	May regulate the activity of kinases such as PKC and SRC via binding to integrin beta-1 (ITB1) and the receptor of activated protein kinase C (RACK1/GNB2L1).
Platelet-activating factor acetylhydrolase IB subunit beta	P68402	22.7	6.05	2.93	232.12	16.59	3	Inactivates platelet activating factor by removing the acetyl group at the sn-2 position. This is a catalytic subunit.
Hippocalcin-like protein 1	P37235	20.3	5.68	2.92	1693.38	81.87	15	May be involved in the calcium-dependent regulation of rhodopsin phosphorylation and may be of relevance for neuronal signalling in the central nervous system
Protein S-100A6	P06703	10.2	5.48	1.70	567.83	45.56	3	This protein may function in stimulation of Ca2+-dependent insulin release, stimulation of prolactin secretion, and exocytosis
Cofilin-1	P23528	18.5	8.09	2.0	2365.53	84.34	17	Important for normal progress through mitosis and normal cytokinesis. It depolymerizes filamentous F-actin and inhibits the polymerization of monomeric G-actin in a pH-dependent manner.
Calretinin	P22676	31.5	5.15	1.75	1217.24	53.14	15	Calretinin is a calcium-binding protein which is abundant in auditory neurons
Chloride intracellular channel protein 4	Q9Y696	28.8	5.34	2.0	1261.45	59.29	13	Chloride channels are a diverse group of proteins that regulate fundamental cellular processes including stabilization of cell membrane potential, transepithelial transport, maintenance of intracellular pH, and regulation of cell volume
26S proteasome non-ATPase regulatory subunit 14	O00487	34.6	6.52	1.87	1112.6	47.74	9	Metalloprotease component of the 26S proteasome that specifically cleaves ‘Lys-63’-linked polyubiquitin chains. Plays a role in response to double-strand breaks.
Annexin A1	P04083	38.7	7.02	2.50	2360.7	64.16	21	Calcium/phospholipid-binding protein which promotes membrane fusion and is involved in exocytosis. This protein regulates phospholipase A2 activity.
LIM and SH3 domain protein 1	Q14847	29.7	7.05	2.0	302.57	23.37	6	Plays an important role in the regulation of dynamic actin-based, cytoskeletal activities.
Macrophage-capping protein	P40121	38.5	6.19	1.91	392.18	18.10	5	Reversibly blocks the barbed ends of F-actin filaments in a Ca2+ and phosphoinositide-regulated manner, but does not sever preformed actin filaments. By capping the barbed ends of actin filaments, the encoded protein contributes to the control of actin-based motility in non-muscle cells.
Calponin-3	Q15417	36.4	6.05	2.0	26.83	37.81	8	Implicated in the regulation and modulation of smooth muscle contraction. It is capable of binding to actin, calmodulin, troponin C and tropomyosin. The interaction of calponin with actin inhibits the actomyosin Mg-ATPase activity.
Rab GDP dissociation inhibitor beta	P50395	51.1	6.47	1.92	948.84	53.93	21	Regulates the GDP/GTP exchange reaction of most Rab proteins by inhibiting the dissociation of GDP from them, and the subsequent binding of GTP to them.
Tubulin alpha-1B chain	P68363	57.7	5.07	5.52	2013.84	54.77	17	Tubulin is the major constituent of microtubules. It binds two moles of GTP, one at an exchangeable site on the beta chain and one at a non-exchangeable site on the alpha chain.
T-complex protein 1 subunit theta	P50990	59.6	5.39	4.62	533.2	59.55	35	Molecular chaperone; assists the folding of proteins upon ATP hydrolysis. As part of the BBS/CCT complex may play a role in the assembly of BBSome, a complex involved in ciliogenesis regulating transports vesicles to the cilia. Known to play a role, in vitro, in the folding of actin and tubulin
Dihydrolipoyllysine-residue acetyltransferase component of pyruvate dehydrogenase complex, mitochondrial	P10515	69	7.84	1.9	2914.38	43.89	21	The pyruvate dehydrogenase complex catalyzes the overall conversion of pyruvate to acetyl-CoA and CO2, and thereby links the glycolytic pathway to the tricarboxylic cycle.
Ubiquilin-2	Q9UHD9	65.7	5.22	1.71	516.53	22.28	8	Increases the half-life of proteins destined to be degraded by the proteasome; may modulate proteasome-mediated protein degradation.
Stress-70 protein, mitochondrial	P38646	73.6	5.94	1.73	4036.24	55.96	34	Implicated in the control of cell proliferation and cellular aging. May also act as a chaperone
Cold shock domain-containing protein E1	O75534	90.5	6.48	1.77	2079.09	44.86	34	Required for internal initiation of translation of human rhinovirus RNA. May be involved in translationally coupled mRNA turnover. Implicated with other RNA-binding proteins in the cytoplasmic deadenylation/translational and decay interplay of the FOS mRNA mediated by the major coding-region determinant of instability (mCRD) domain
Vinculin	P18206	110.2	6.34	2.0	4523.83	58.11	61	Actin filament (F-actin)-binding protein involved in cell-matrix adhesion and cell-cell adhesion. Regulates cell-surface E-cadherin expression and potentiates mechanosensing by the E-cadherin complex. May also play important roles in cell morphology and locomotion
Leucine-rich PPR motif-containing protein, mitochondrial	P42704	157.8	6.13	2.0	4844.96	59.33	74	May play a role in RNA metabolism in both nuclei and mitochondria. In the nucleus binds to HNRPA1-associated poly(A) mRNAs and is part of nmRNP complexes at late stages of mRNA maturation which are possibly associated with nuclear mRNA export
Vimentin	P08670	57	5.6	AT	1938.13	42.49	27	Vimentins are class-III intermediate filaments found in various non-epithelial cells, especially mesenchymal cells. Vimentin is attached to the nucleus, endoplasmic reticulum, and mitochondria, either laterally or terminally
Transforming protein RhoA	P61586	14.7	4.93	AT	1717.95	81.35	13	Regulates a signal transduction pathway linking plasma membrane receptors to the assembly of focal adhesions and actin stress fibers
Vimentin	P08670	57	5.6	AT	2054.84	59.23	27	
Vimentin	P08670	57	5.6	AT	1584.79	36.70	19	
Adenine phosphoribosyltransferase	P07741	19.6	5.59	2.42	1162.47	71.67	9	Catalyzes a salvage reaction resulting in the formation of AMP, that is energically less costly than de novo synthesis
Calumenin	O43852	37.1	4.64	1.89	2462.85	71.43	23	Involved in regulation of vitamin K-dependent carboxylation of multiple N-terminal glutamate residues
SH3 domain binding glutamic acid-rich protein like 3	Q9H299	10.4	4.93	1.85	698.5	39.78	4	Could act as a modulator of glutaredoxin biological activity
Translationally-controlled tumor protein	E9PJF7	18.2	4.79	1.97	277.25	30.86	9	TCTP functions as molecule that prevents cell death. It prevents cell death by binding to calcium, an ion that causes cell death. Furthermore, the N-terminal domain of TCTP inhibits apoptosis by binding to apoptotic factors and by inhibiting p53 tumour suppressor-dependent apoptosis by downregulating it.
Translationally-controlled tumor protein	P13693	19.6	4.93	1.78	864.91	51.16	8	TCTP functions as molecule that prevents cell death. It prevents cell death by binding to calcium, an ion that causes cell death. Furthermore, the N-terminal domain of TCTP inhibits apoptosis by binding to apoptotic factors and by inhibiting p53 tumour suppressor-dependent apoptosis by downregulating it.
14-3-3 protein gamma, N-terminally processed	P61981	28.3	4.89	1.99	1153.87	62.75	14	Adapter protein implicated in the regulation of a large spectrum of both general and specialized signaling pathways. Binds to a large number of partners, usually by recognition of a phosphoserine or phosphothreonine motif.
Actin, cytoplasmic 1	P60709	37.4	5.58	1.8	43.27	40.24	10	Actins are highly conserved proteins that are involved in various types of cell motility and are ubiquitously expressed in all eukaryotic cells.
Elongation factor 1-delta	P29692	31.1	5.01	2.21	1567.43	53.3	15	Isoform 1: EF-1-beta and EF-1-delta stimulate the exchange of GDP bound to EF-1-alpha to GTP, regenerating EF-1-alpha for another round of transfer of aminoacyl-tRNAs to the ribosome. Isoform 2:Regulates induction of heat-shock-responsive genes through association with heat shock transcription factors and direct DNA-binding at heat shock promoter elements (HSE)
Elongation factor 1-delta	P29692	31.1	5.01	1.82	1776.28	64.06	18	
Aldehyde dehydrogenase X, mitochondrial	P30837	57.2	6.8	2.58	2153.09	41.01	21	ALDHs play a major role in the detoxification of alcohol-derived acetaldehyde. They are involved in the metabolism of corticosteroids, biogenic amines, neurotransmitters, and lipid peroxidation
Tubulin alpha-1C	Q9BQE3	57.7	5.07	1.92	123.2	43.35	7	Tubulin is the major constituent of microtubules
Calcium-binding and coiled-coil domain-containing protein	Q13137	54.6	5.02	2.0	55.45	34.05	15	May play a role in ruffle formation and actin cytoskeleton organization
Sec1 family domain containing 1	Q8WVM8	72.3	6.27	2.17	2764.17	59.35	27	Plays a role in SNARE-pin assembly and Golgi-to-ER retrograde transport via its interaction with COG4. Involved in vesicular transport between the endoplasmic reticulum and the Golgi

Database IPI human v3.12; MS/MS ion search, peptide mass tolerance: ±10 ppm, fragment mass tolerance: ±0.8 Da, enzyme: trypsin, variable modifications: carbamidomethyl (C), oxidation (M), max missed cleavages: 2; Appeared upon treatment (AT).

### Hierarchial clustering, Gene Ontology (GO) and Network analysis classification of differentially regulated protein

**Figure 4:**
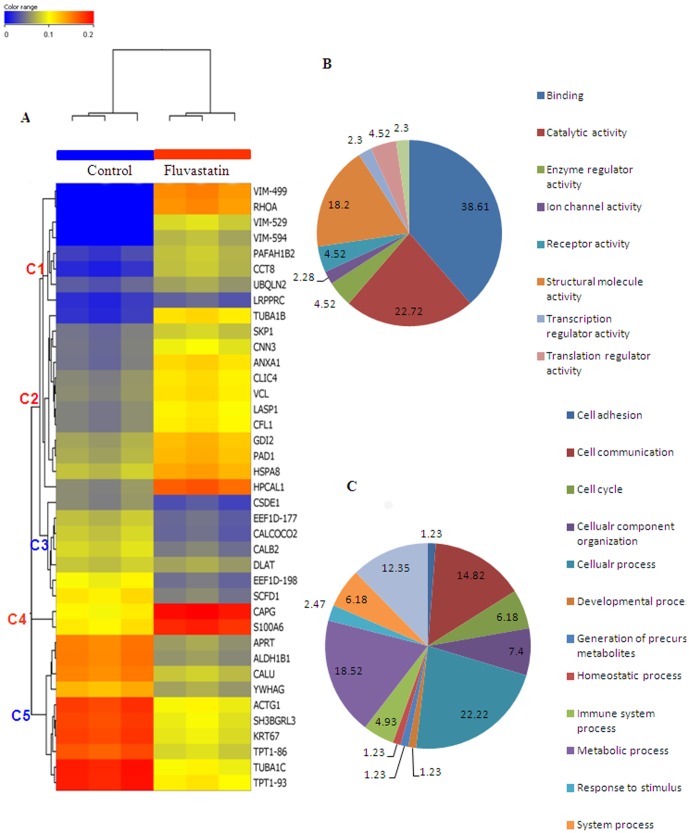
Heat map and and Gene Ontology analysis of fluvastatin mediated proteome profile in MDA-MB-231 cells. A, represents the heat map of all the differentially regulated proteins generated by Agilent's GeneSpring GX 11.0. B represents molecular function and C, shows biological processes of differentially regulated proteins as deciphered by gene ontology analysis. The data labels shown on the pie chart represents % proteins involved in a particular molecular function (A) or biological process (B).

**Figure 5:**
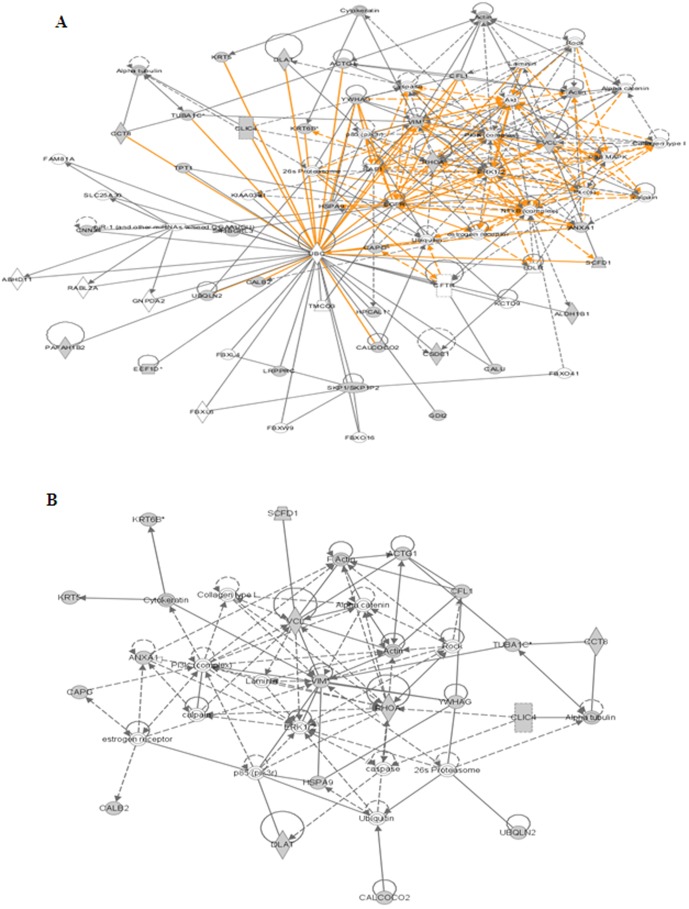
Protein sub networks of fluvastatin mediated differentially regulated proteins. Protein-protein physical/functional interaction global complex network (A) and sub network (B) generated by Ingenuity Pathway Analysis tool. Grey filled boxes are the differentially expressed proteins. Only significant sub networks are represented.

To understand the patterns of differentially regulated proteins, unsupervised hierarchical clustering has been performed. From the presented heat map (Fig. 4A), we identified one major (C1) and two (C2&C4) minor clusters as up regulated and two major clusters (C3&C5) comprising of down regulated proteins. However, this analysis showed that none of these protein clusters represented any of the particular biological pathway or molecular functions related to cancer but nonetheless, protein clusters represented multiple pathways and cellular functions. Therefore to further understand the role and relevance of different proteins that are precisely involved in statin mediated breast cancer cell death, all the differentially regulated proteins were mapped to various biological networks using IPA analysis. DAVID GO annotation analysis identified that most of the proteins that were differentially regulated between fluvastatin and untreated cells clustered in biological processes and molecular functions regulating cell adhesion, cell communication and cell cycle (Fig. 4B and 4C). In addition to the GO analysis, protein network analysis was computed using the Ingenuity Pathways Analysis (IPA) tool and the list of most significant networks is presented in Table 2. This analysis showed that the networks presented in Fig. 5A were significantly altered in MDA-MB-231 cells up on exposure to 10 µM fluvastatin for a period of 24 h. Global proteome network of fluvastatin-mediated differentially regulated proteins directly or indirectly are linked with many protein hubs that are highly interconnected to RhoA, Akt, ERK1/2, PI3K, NF-kB complex, vimentin, ubiquitin C and 26S proteasome networks, which are known to be involved in controlling cell proliferation. From sub network analysis as shown in Fig. 5B, it was observed that the altered cytoskeletal proteins are highly interconnected through vimentin as a central node.

**Table 2:** IPA analysis of fluvastatin mediated differentially regulated proteins.

S. No	Significant networks
1	Cell-To-Cell Signaling and Interaction, Cellular Movement, Hematological System Development and Function
2	Organismal Injury and Abnormalities, Digestive System Development and Function, Endocrine System Development and Function
	**Molecular and Cellular Functions**
1	Cell-To-Cell Signaling and Interaction
2	Cellular Movement
3	Cellular Compromise
4	Cellular Function and Maintenance
5	Cell Morphology
	**Top Canonical Pathways**
1	Epithelial Adherens Junction Signaling
2	Remodeling of Epithelial Adherens Junctions
3	RhoGDI Signaling
4	ILK Signaling
5	Actin Cytoskeleton Signaling

The most significant networks, pathways and molecular functions are summarized.

### Fluvastatin cause dysregulation of vimentin, a major cytoskeletal protein in MDA-MB-231 cells: Effect of mevalonate

**Figure 6:**
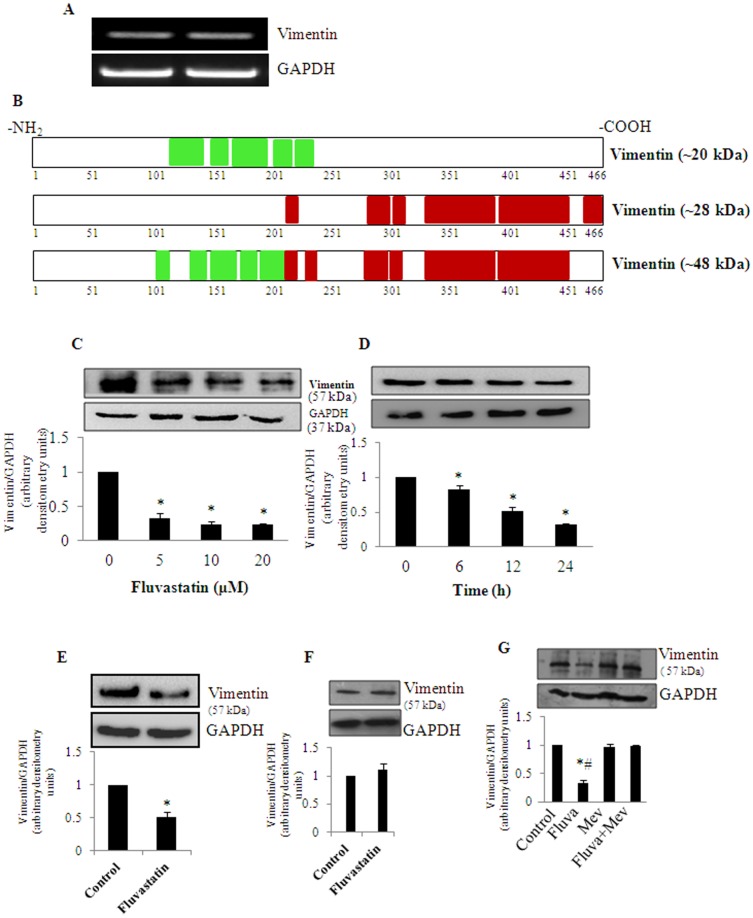
Fluvastatin causes controlled proteolysis of vimentin in breast cancer cells. A, shows the transcript levels of vimentin in presence and absence of fluvastatin (10 µM) for 24 h in MDA-MB-231 cells. B, shows the identified peptide sequence coverage of two different MW's of truncated vimentin protein spots appeared in fluvastatin (10 µM) treated MDA-MB-231 cells for 24 h. C, MDA-MB-231 cells were treated with various concentrations of statin (0–20 µM) for a period of 24 h and at the end of the treatments, vimentin protein levels were measured by Western blot analysis. D is same as C except that cells were treated with fluvastatin (10 µM) for various time points (0–24 h) and vimentin protein levels were measured by Western analysis. E, BT-549 cells were treated with fluvastatin (20 µM) for 24 h and vimentin levels were measured by Western blot analysis. F, MCF-10A cells were either untreated or treated with fluvastatin (10 µM) for a period of 24 h and vimentin protein levels were measured by Western analysis. G, MDA-MB-231 cells were treated in presence or absence of mevalonate (25 µM) for a period of 24 h and vimentin protein levels were measured by Western analysis. Data represented are Mean±SD from at least three independent experiments. *, significantly different compared to untreated conditions; #, significantly different compared to mevalonate and fluva+mev condition. Statistical significance was tested at P<0.05 level by two-tailed, unpaired, Student's t-test.

Notably, from the network analysis and GO results it was observed that the cytoskelatal protein vimentin formed a major hub connecting other differentially regulated proteins directly or indirectly. Intriguingly, our proteomic data revealed that vimentin was identifed in 2D gel spots at three different pI's and molecular weight regions. To gain more insights on this discrepancy, we then measured mRNA levels of vimentin using full length primers to see if there are any alterative spliced isoforms of vimentin in untreated and fluvastatin treated MDA-MB-231 cells. However, we could not detect multiple gene products of vimentin which indicates that vimentin has no alternatively spliced isoforms in MDA-MB-231 cells (Fig. 6A). Next, to see the possibility of the involvement of proteolysis playing a role in the degradation of vimentin and thereby leading to the appearance of the observed 3 spots with low molecular weight (∼48, ∼28 and ∼20 kDa) compared to full length protein (∼57 kDa), we have analyzed the tryptic peptides identifiedby mass spectrometry. Based on the MS-MS analysis, it appears that vimentin is truncated at either N-terminal or C-terminal regions leading to the generation of two spots spanning from amino acids 101–236 and 207–466 respectively with different molecular weights as seen in the 2D gel separation (Fig. 6B). The other spot (with observed molecular weight of ∼48 kDa) was identified from peptides spanning from N-terminal to C-terminal region. These observations suggest that vimentin undergoes controlled proteolysis after fluvastatin treatment in MDA-MB-231 cells. In the vimentin sub network, we have also observed that many of the cytoskeletal and their regulatory proteins such as vinculin, actin γ1, cofilin and tubulin α 1C are directly or indirectly connected to the regulation of vimentin. This suggests that vimentin may play a pivotal role in fluvastatin mediated cytotoxicity in MDA-MB-231 breast cancer cells. Furthermore, to validate the altered regulation of vimentin with fluvastatin treatment that was identified with 2DE coupled MS-MS analysis; we have checked its levels by Western blot analysis. For this, cells were treated with various concentrations of fluvastatin (0–20 µM) as a function of time. The results showed that fluvastatin dose- and -time dependently decreased vimentin levels (Fig. 6C and 6D). Similar results were observed in BT-549 cells (Fig. 6E). These results are in agreement with the altered vimentin levels seen in proteomic studies upon fluvastatin treatment. On the other hand, interestingly, fluvastatin did not affect vimentin levels in normal mammary epithelial (MCF-10A) cells (Fig. 6F)wherein, fluvastatin did not induce any appreciable cell death (Fig. 1C). This result indicates that fluvastatin exerts distinct effects in normal and cancer cells. To determine whether the statin-induced decrease in vimentin is mediated through HMG-CoAR inhibition, MDA-MB-231 cells were co-treated with fluvastain (10 µM) in the presence or absence of mevalonate (25 µM) for 24 h. It was found that mevalonate significantly restored fluvastatin-mediated down regulation of vimentin levels (Fig. 6G). This result implicate that mevalonate down stream products are responsible for regulating vimentin and a reduction in vimentin levels may in part contribute to fluvastatin-mediated cell death in MDA-MB-231 cells.

### Caspase-3 but not proteasomal activation down regulates vimentin levels in MDA-MB-231 cells

**Figure 7:**
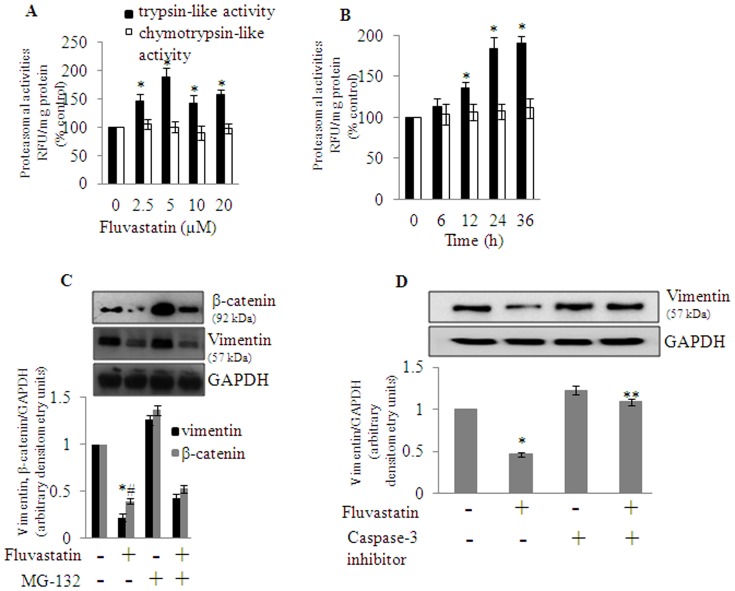
Caspase-3 inhibitor but not proteasomal inhibitor alters fluvastatin-mediated regulation of vimentin levels in MDA-MB-231 cells. A, MDA-MB-231 cells were treated with various concentrations of fluvastatin (0–20 µM) for a period of 24 h and at the end of the treatments trypsin-like and chymotrypsin-like activities of the 26S proteasome were measured using respective fluorogenic substrates as described in materials and methods section. B, is same as A except that cells were treated with fluvastatin (10 µM) for various time points (0–24 h). C, MDA-MB-231 cells were treated in the presence or absence of MG-132 (1 µM) for a period of 24 h and at the end of the treatments, vimentin and β-catenin protein levels were measured by Western blot analysis. D, MDA-MB-231 cells were pre-treated with caspase-3 inhibitor (50 µM) for a period of 6 h in the presence or absence of fluvastatin (10 µM) for a period of 24 h and vimentin protein levels were measured by Western analysis. Data represented are Mean±SD from at least three independent experiments. *, significantly different compared to untreated conditions; #, significantly different compared to MG-132 and fluva+MG-132 condition; **, significantly different compared to fluvastatin alone treated condition. Statistical significance was tested at P<0.05 level by two-tailed, unpaired, Student's t-test.

To gain more insights into the statin-mediated dysregulation of vimentin levels through increased proteolysis, we initially measured 26S proteasomal activities (both trypsin-like and chymotrypsin-like) in cells treated with various concentrations (0–20 µM) of fluvastatin for 24 h. I Fluvastatin dose-dependently increased the trypsin-like activity of the 26S proteasome from 0–5 µM of fluvastatin and tapered off from there (Fig. 7A). However, the chymotrpsin-like activity was unchanged under the same experimental conditions (Fig. 7A). Fluvastatin started to show a significant activation of trypsin-like activity by 12 h and reached peak by 24 h (Fig. 7B). Next, to see if the increased proteasomal activity was responsible for the regulation of vimentin in fluvastatin treated cells, we measured both β-catenin, an upstream regulator of vimentin in the presence or absence of MG-132 (proteasomal inhibitor). Results showed that fluvastatin-mediated down-regulation of β-catenin was significantly reversed in the presence of MG-132 (Fig. 7C). Treatment of cells with MG-132 alone greatly accumulated β-catenin even when compared to untreated conditions (Fig. 7C). However, under the same experimental conditions, to our surprise, vimentin levels were unchanged with MG-132 treatment (Fig. 7C). This result suggests that an increased level of β-catenin due to proteasomal inhibition per se was not responsible for the regulation of vimentin in fluvastatin treated MDA-MB-231 cells. To resolve this discrepancy, we then went on to check the effect of caspase-3 inhibitor in regulating vimentin levels because we recently reported that fluvastatin treatment dose- and time-dependently increased caspase-3 like protease activity in TNBC cells like MDA-MB-231, MDA-MB-453 and BT-549 cells [7]. Interestingly, caspase-3 inhibitor, Boc-D-FMK (50 µM) treated for 12 h in the presence of fluvastatin (5 µM) greatly rescued the fluvastatin-mediated down regulation of vimentin (Fig. 7D). Thereby suggesting that caspase-3 activity but not 26S proteasomal activity regulates vimentin levels in these cells under fluvastatin treated conditions.

## Discussion

A very recent study showed that the basal type breast cancers are highly sensitive to fluvastatin and it is negatively associated with expression of estrogen receptor alpha (ERα) [15]. Though different mechanisms have been proposed for the anti cancer potential of statins, information is still limited on the global proteome or gene expression changes associated with statin treatment to identify possible molecular signatures responsible for their anti cancer properties. Hence, in the present study we focussed on a proteome analysis of MDA-MB-231, a triple negative metastatic breast cancer cell type challenged with fluvastatin to determine the protein alterations associated with this treatment employing 2DE coupled mass spectrometry. Our proteomic analysis data showed differential expression of 39 spots representing 35 different proteins in MDA-MB-231 cells treated with fluvastatin compared to untreated conditions. The identified proteins represent various biological functions known in different cancers. Lipophilic statins are effective against triple negative breast cancer and by and large; it appears that different lipophilic statins induce a similar response in mediating anti-cancer effects. In an earlier study with lovastatin, it was shown that GTPase mediated cell signaling proteins like CDC42, pG3PI1 were up regulated upon lovastatin treatment but the changes in these proteins were not found in our study with fulvastatin treatment. On the other hand, an up regulation of RhoA and GDI2 was seen in both studies. The difference between the earlier study with lovastatin and the current study with fluvastatin is that, in the former study, MDA-MB-231 cells were treated with 20 µM lovastatin for a period of 48 h and in the current study, cells were treated with 10 µM fluvastatin for a period of 24 h. Nevertheless, we have observed ANXA1, CLIC, CFL1, EEF1D, CAPG, RhoA and VCL proteins are being regulated commonly by either lovastatin or fluvastatin [9]. In search of the molecular functions and biological processes involved with the list of identified proteins by 2DE coupled mass spectrometry, we adapted Gene Ontology (GO) approach [16]. Using GO, these proteins were classified into several groups related to the regulators of cell proliferation, migration and apoptosis. Many of these altered proteins are involved in binding and within this class, majority of them are enriched with calcium ion binding and GTPase activity protein binding. Small G-proteins regulate many cellular functions including cytoskeletal rearrangement, cell motility, intracellular trafficking, transcriptional regulation, cell growth, and development [17]. It is possible that statins by depleting isoprenoids may alter many of the cytoskeletal protein functions and disrupts cell morphology. Statins are known to involve in inhibiting cancer cell proliferation by arresting the cell cycle at the G0/G1 phase and induce apoptosis [6], [18]. To corroborate this, GO analysis showed that cell cycle regulatory proteins such as SKP1 and S-100A6 were up regulated with fluvastatin treatment which in turn have a role in inducing apoptosis in breast cancer cells.

Furthermore, mapping of the differentially expressed proteins to biological networks curated in database Ingenuity pathway Analysis (IPA) has identified several significant subnet works. Among the networks built from the current analysis, most significant sub network formed with vimentin as a central node connecting to several sub nodes. This interesting observation suggests a possible role for vimentin in statin mediated cell death. Vimentin, an epithelial- mesenchymal transition marker, is elevated in various epithelial cancers including breast cancer and recently it was also highlighted as a potential therapeutic target for cancer because of its role in proliferation, migration and invasion [19], [20]. Further, it was reported that loss of cytokeratin and gain of vimentin expression are indicators of biologically aggressive breast carcinoma [21]. Vimentin, Zeb1 and Sip1 are high in TNBC compared to non-TNBC cells and it was also shown that over expression of vimentin is associated with poor prognosis of breast cancer [22]. Up on treatment with fluvastatin, vimentin was down regulated in MDA-MB-231 breast cancer cells by Western blot analysis. With 2DE coupled LC-MS/MS approach, we identified three vimentin protein spots corresponding to approximately 20 kDa, 28 kDa and 48 kDa in fluvastatin treated MDA-MB-231 cells. These multiple spots of vimentin were not seen in untreated cells. This result indicates that vimentin possibly undergoes a controlled proteolytic degradation during fluvastatin treatment as evidenced by the sequence coverage of tryptic peptides which supported truncated fragments of vimentin. In the presence of mevalonate, an immediate downstream product of HMGCoA, fluvastatin mediated down regulation of vimentin was significantly reversed, thereby suggesting a possible role for vimentin in cell survival and proliferation of breast cancer cells. Surprisingly, vimentin levels were not altered upon fluvastatin in normal mammary epithelial cells indicating its active role in cancer cell proliferation. Previously it was reported that preferential cleavage of vimentin at Asp85 by caspase-3/7 and Asp259 by caspase-6 yields truncated fragments and further dismantles intermediate filaments. Furthermore, proteolysis of vimentin at Asp85 generates a pro apoptotic amino terminal signal that assists in further degradation of vimentin and induction of apoptosis in HeLa cells [23]. Along these lines, we have also recently reported that fluvastatin induces caspase activity in breast cancer cells [7]. In the present study, we found that statin mediated down regulation of vimentin was rescued in presence of caspase-3 inhibitor (BOC-D-FMK). Therefore, it is likely that fluvastatin induce proteolysis of vimentin at Asp85, which in turn facilitates apoptosis in MDA-MB-231 cells by activating caspase-mediated proteolysis. Vimentin, apart from its cytoskeletal regulation, has been identified as a downstream target of major cell signaling cascades including ERK, AKT, STAT3 and PI3K [24]–[26]. Hence, statins by altering these signaling pathways may accelerate vimentin proteolysis which in turn disrupts the cellular organization, reduces metastasis and induce apoptosis.

Recently, it was reported that simvastatin in combination with irinotecan induces apoptosis and cell cycle arrest by inhibiting the proteasome activity in A-549, NCI-H460 human non-small cell lung cancer cells [27]. Furthermore, farnesyl and geranyl geranyl transferase inhibitors and other agents containing active lactone moiety arrest cells at G1stage by inhibiting proteasome and upregulation of p21 in HS578-T, MDA-MB-231 and MDA-MB-436 breast cancer cells [28]. In contrast to this, it was also reported that statins (both lipophilic and hydrophilic) did not alter the 20s proteasome activity in mammalian endothelial and vascular smooth muscle cells [29]. This difference may be due to the different cell types employed in those studies i.e., transformed *vs* normal cells. Along these lines, in this study we were interested in understanding the involvement of proteasomal enzyme activities in fluvastatin-mediated MDA-MB-231 cell death and also their relevance in the regulation of vimentin proteolysis. We observed that fluvastatin increases trypsin-like but not chymotrypsin-like proteosomal activity of the 26S proteasome. But to our surprise, fluvastatin-mediated vimentin down regulation was not rescued in presence of MG-132, a proteasomal inhibitor. This suggests fluvastatin-induced vimentin degradation is not mediated by proteasomal enzymes. It is known that the vimentin promoter is a direct target of β-catenin/TCF transactivation in breast cancer cells and the cytoplasmic and nuclear localization of β-catenin in mammary human epithelial cell lines is associated with vimentin expression leading to high invasive/migratory potential [19]. Interestingly, in the present study it was found that β-catenin, an upstream regulator of vimentin was significantly down regulated with fluvastatin treatment which was reversed in the presence of MG-132. However, this reversal was not seen with vimentin levels. Treatment of MG-132 alone to MDA-MB-231 cells greatly accumulated β-catenin indicating that β-catenin but not vimentin is regulated by proteasomal machinery. This clearly indicates that β-catenin is not the only upstream regulator of vimentin in MDA-MB-231 cells. It is known that Smad interacting protein-1(SIP1) directly regulates vimentin expression in human mammary epithelial (MCF-10A) cells and also the activation of the vimentin promoter by SIP1 was not associated with the activation of the β-catenin/TCF/LEF signaling pathway [30]. Therefore, it is tempting to speculate that SIP1 regulates vimentin levels in MDA-MB-231 cells. Apart from vimentin, translationally controlled tumor protein (TPT1) and elongation factor 1-δ (EEF1D) are among the statin mediated down regulated proteins that were identified as multiple protein spots in 2D-gel. Interestingly, TPT1/TCTP protein is known to act as anti apoptotic protein in cancer cells and is highly expressed in various cancers including breast cancer. Recently it was also reported that high-TPT1 status associates with poorly differentiated, aggressive grade breast tumors, predicting poor prognosis [31]. Similarly, Elongation factor-1 (EF-1) delta is one of the four subunits of elongation factor-1 complex involved in elongation step during the protein synthesis. Previously it was shown that EF-1 delta mRNA expression was significantly higher in cancerous compared to noncancerous oesophageal tissues, and a higher expression of EF-1 delta mRNA was correlated with lymph node metastases, advanced disease stages and poorer prognosis for patients with oesophageal carcinoma [32].

## Conclusions

The present study reports that fluvastatin induces breast cancer cell death by altering various cellular proteins. Among the differentially regulated proteins, three proteins were observed at more than one spot identified as vimentin, translationally controlled tumor protein and eukaryotic elongation factor 1 delta. Perhaps for the first time, we show that statins target vimentin by inducing caspase mediated proteolysis. Vimentin proteolysis was rescued in the presence of mevalonate and may play a role in protecting from statin-induced breast cancer cell death as vimentin expression was not altered in normal mammary epithelial cells. Importantly, statin mediated vimentin down regulation and other differentially regulated proteins may be used as biomarkers upon further validation in TNBC patients.

## References

[1] Bjarnadottir O, Romero Q, Bendahl PO, Jirstrom K, Ryden L, et al (2013) Targeting HMG-CoA reductase with statins in a window-of-opportunity breast cancer trial. Breast Cancer ResTreat 138: 499–508. 10.1007/s10549-013-2473-6 23471651

[2] Demierre MF, Higgins PD, Gruber SB, Hawk E, Lippman SM (2005) Statins and cancer prevention. Nat Rev Cancer 5: 930–42. 16341084 10.1038/nrc1751

[3] Garwood ER, Kumar AS, Baehner FL, Moore DH, Au A, et al (2010) Fluvastatin reduces proliferation and increases apoptosis in women with high grade breast cancer. Breast Cancer ResTreat 119: 137–44. 10.1007/s10549-009-0507-x PMC4087110 19728082

[4] Nielsen SF, Nordestgaard BG, Bojesen SE (2013) Statin use and reduced cancer-related mortality. N Engl J Med 368: 576–7. 10.1056/NEJMc1214827 23388012

[5] Campbell MJ, Esserman LJ, Zhou Y, Shoemaker M, Lobo M, et al (2006) Breast cancer growth prevention by statins. Cancer Res 66: 8707–14. 16951186 10.1158/0008-5472.CAN-05-4061

[6] Kotamraju S, Williams CL, Kalyanaraman B (2007) Statin-induced breast cancer cell death: role of inducible nitric oxide and arginase-dependent pathways. Cancer Res 67: 7386–94. 17671209 10.1158/0008-5472.CAN-07-0993

[7] Kanugula AK, Gollavilli PN, Vasamsetti SB, Karnewar S, Gopoju R, et al (2014) Statin-induced inhibition of breast cancer proliferation and invasion involves attenuation of iron transport: Intermediacy of nitric oxide and antioxidant defence mechanisms. FEBS J 281: 3719–38. 24964743 10.1111/febs.12893

[8] Aka JA, Lin SX (2012) Comparison of functional proteomic analyses of human breast cancer cell lines T47D and MCF7. PLoS One 7: e31532. 22384035 10.1371/journal.pone.0031532 PMC3286449

[9] Klawitter J, Shokati T, Moll V, Christians U, Klawitter J (2010) Effects of lovastatin on breast cancer cells: a proteo-metabonomic study. Breast Cancer Res 12: R16. 20205716 10.1186/bcr2485 PMC2879560

[10] Shui HA, Hsia CW, Chen HM, Chang TC, Wang CY (2012) Proteomics and bioinformatics analysis of lovastatin-induced differentiation in ARO cells. J Proteomics 75: 1170–80. 22086082 10.1016/j.jprot.2011.10.029

[11] Leong S, Nunez AC, Lin MZ, Crossett B, Christopherson RI, et al (2012) iTRAQ-based proteomic profiling of breast cancer cell response to doxorubicin and TRAIL. J Proteome Res 11: 3561–72. 22587632 10.1021/pr2012335

[12] Dong X, Xiao Y, Jiang X, Wang Y (2011) Quantitative proteomic analysis revealed lovastatin-induced perturbation of cellular pathways in HL-60 cells. J Proteome Res 10: 5463–71. 21967149 10.1021/pr200718p PMC3230662

[13] Ummanni R, Junker H, Zimmermann U, Venz S, Teller S, et al (2008) Prohibitin identified by proteomic analysis of prostate biopsies distinguishes hyperplasia and cancer. Cancer Lett 266: 171–85. 18384941 10.1016/j.canlet.2008.02.047

[14] Kotamraju S, Tampo Y, Keszler A, Chitambar CR, Joseph J, et al (2003) Nitric oxide inhibits H2O2-induced transferrin receptor-dependent apoptosis in endothelial cells: Role of ubiquitin-proteasome pathway. Proc Natl Acad Sci USA 100: 10653–8. 12958216 10.1073/pnas.1933581100 PMC196859

[15] Goard CA, Chan-Seng-Yue M, Mullen PJ, Quiroga AD, Wasylishen AR, et al (2014) Identifying molecular features that distinguish fluvastatin-sensitive breast tumor cells. Breast Cancer Res Treat 143: 301–12. 24337703 10.1007/s10549-013-2800-y

[16] Thomas PD, Campbell MJ, Kejariwal A, Mi H, Karlak B, et al (2003) PANTHER: a library of protein families and subfamilies indexed by function. Genome Res 13: 2129–41. 12952881 10.1101/gr.772403 PMC403709

[17] Takai Y, Sasaki T, Matozaki T (2001) Small GTP-binding proteins. Physiol Rev 81: 153–208. 11152757 10.1152/physrev.2001.81.1.153

[18] Sivaprasad U, Abbas T, Dutta A (2006) Differential efficacy of 3-hydroxy-3-methylglutaryl CoA reductase inhibitors on the cell cycle of prostate cancer cells. Mol Cancer Ther 5: 2310–6. 16985065 10.1158/1535-7163.MCT-06-0175

[19] Gilles C, Polette M, Mestdagt M, Nawrocki-Raby B, Ruggeri P, et al (2003) Transactivation of vimentin by beta-catenin in human breast cancer cells. Cancer Res 63: 2658–64. 12750294

[20] Satelli A, Li S (2011) Vimentin in cancer and its potential as a molecular target for cancer therapy. Cell Mol Life Sci 68: 3033–46. 21637948 10.1007/s00018-011-0735-1 PMC3162105

[21] Vora HH, Patel NA, Rajvik KN, Mehta SV, Brahmbhatt BV, et al (2009) Cytokeratin and vimentin expression in breast cancer. Int J Biol Markers 24: 38–46. 19404921 10.1177/172460080902400106

[22] Karihtala P, Auvinen P, Kauppila S, Haapasaari KM, Jukkola-Vuorinen A, et al (2013) Vimentin, zeb1 and Sip1 are up-regulated in triple-negative and basal-like breast cancers: association with an aggressive tumour phenotype. Breast Cancer Res Treat 138: 81–90. 23412770 10.1007/s10549-013-2442-0

[23] Byun Y, Chen F, Chang R, Trivedi M, Green KJ, et al (2001) Caspase cleavage of vimentin disrupts intermediate filaments and promotes apoptosis. Cell Death Differ 8: 443–50. 11423904 10.1038/sj.cdd.4400840

[24] Barberis L, Pasquali C, Bertschy-Meier D, Cuccurullo A, Costa C, et al (2009) Leukocyte transmigration is modulated by chemokine-mediated PI3Kgamma-dependent phosphorylation of vimentin. Eur J Immunol 39: 1136–46. 19291697 10.1002/eji.200838884

[25] Perlson E, Michaelevski I, Kowalsman N, Ben-Yaakov K, Shaked M, et al (2006) Vimentin binding to phosphorylated Erk sterically hinders enzymatic dephosphorylation of the kinase. J Mol Biol 364: 938–44. 17046786 10.1016/j.jmb.2006.09.056

[26] Zhu QS, Rosenblatt K, Huang KL, Lahat G, Brobey R, et al (2011) Vimentin is a novel AKT1 target mediating motility and invasion. Oncogene 30: 457–70. 20856200 10.1038/onc.2010.421 PMC3010301

[27] Park IH, Kim JY, Choi JY, Han JY (2011) Simvastatin enhances irinotecan-induced apoptosis in human non-small cell lung cancer cells by inhibition of proteasome activity. Invest New Drugs 29: 883–90. 20467885 10.1007/s10637-010-9439-x

[28] Efuet ET, Keyomarsi K (2006) Farnesyl and geranylgeranyl transferase inhibitors induce G1 arrest by targeting the proteasome. Cancer Res 66: 1040–51. 16424040 10.1158/0008-5472.CAN-05-3416

[29] Ludwig A, Friedel B, Metzkow S, Meiners S, Stangl V, et al (2005) Effect of statins on the proteasomal activity in mammalian endothelial and vascular smooth muscle cells. Biochem Pharmacol 70: 520–6. 15996638 10.1016/j.bcp.2005.04.046

[30] Bindels S, Mestdagt M, Vandewalle C, Jacobs N, Volders L, et al (2006) Regulation of vimentin by SIP1 in human epithelial breast tumor cells. Oncogene 25: 4975–85. 16568083 10.1038/sj.onc.1209511

[31] Amson R, Pece S, Lespagnol A, Vyas R, Mazzarol G, et al (2012) Reciprocal repression between P53 and TCTP. Nat Med 18: 91–9. 10.1038/nm.2546 22157679

[32] Ogawa K, Utsunomiya T, Mimori K, Tanaka Y, Tanaka F, et al (2004) Clinical significance of elongation factor-1 delta mRNA expression in oesophageal carcinoma. Br J Cancer 91: 282–6. 15199388 10.1038/sj.bjc.6601941 PMC2409802

