# Correction: Fluvastatin Mediated Breast Cancer Cell Death: A Proteomic Approach to Identify Differentially Regulated Proteins in MDA-MB-231 Cells

**DOI:** 10.1371/journal.pone.0115568

**Published:** 2014-12-09

**Authors:** The PLOS ONE Staff

The following information is missing from the Funding section: Kanugula acknowledges CSIR (Council of Scientific and Industrial Research) for providing the Senior Research Fellowship. Please view the complete funding statement here.

This work was supported by No.BT/PR9637/BRB/10/582/2007 (http://dbtindia.nic.in/index.asp), and SMiLE-CSC-0111 and EpiHeD-BSC-0118 (http://csirhrdg.res.in/). Kanugula acknowledges CSIR (Council of Scientific and Industrial Research) for providing the Senior Research Fellowship. The funders had no role in study design, data collection and analysis, decision to publish, or preparation of the manuscript.

Additionally, the titles for Figure 3 and Figure 4 are incorrect. Please view the correct titles for Figure 3 and Figure 4 below.

**Figure 3:**
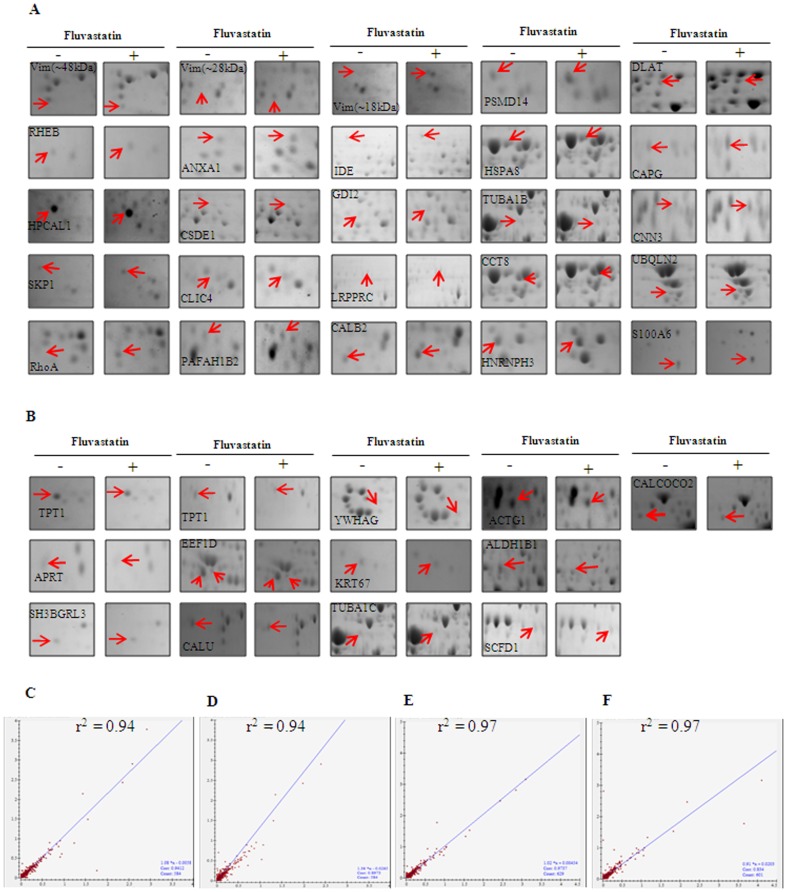
Enlarged view of fluvastatin mediated differentially regulated proteins in MDA-MB-231 cells. A and B shows enlarged view of statin mediated up regulated (A) and down regulated (B) spots respectively. The results presented in C–F are from 3 independent experiments. C and D represent the correlation coefficient of untreated MDA-MB-231 cell lysate. C represents the comparison between gel 1 versus gel 2 and D represents gel 1 versus gel 3. E and F represent the correlation coefficient of statin treated MDA-MB-231 cell lysate. E represents the comparison between gel 1 versus gel 2 and F represents gel 1 versus gel 3.

**Figure 4:**
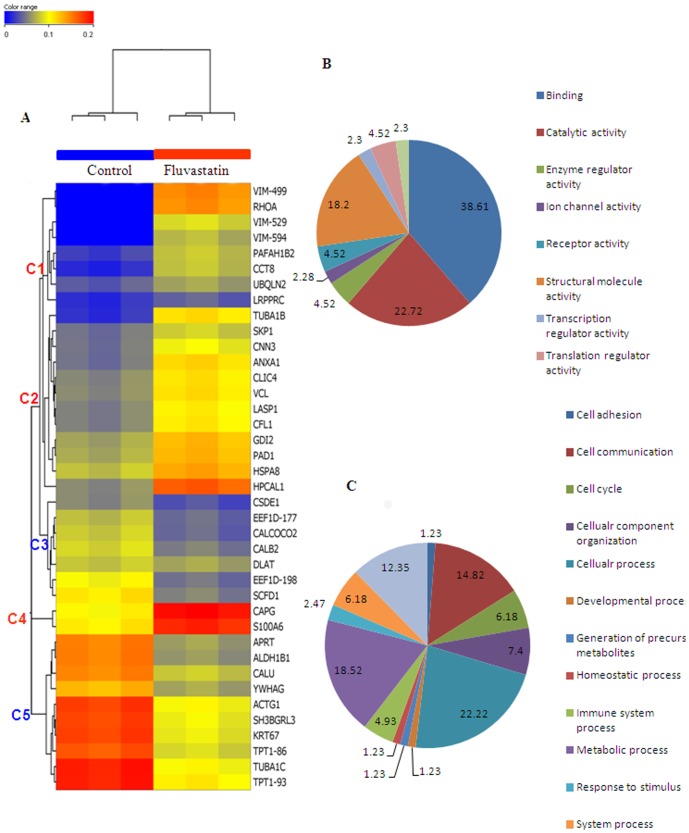
Heat map and Gene Ontology analysis of fluvastatin mediated proteome profile in MDA-MB-231 cells. A, represents the heat map of all the differentially regulated proteins generated by Agilent's GeneSpring GX 11.0. B represents molecular function and C, shows biological processes of differentially regulated proteins as deciphered by gene ontology analysis. The data labels shown on the pie chart represents % proteins involved in a particular molecular function (A) or biological process (B).
